# Isolation and characterization of two new hepatitis E virus genotype 1 strains from two mini-outbreaks in Lahore, Pakistan

**DOI:** 10.1186/1743-422X-8-94

**Published:** 2011-03-04

**Authors:** Tahir Iqbal, Muhammad Idrees, Liaqat Ali, Abrar Hussain, Muhammad Ali, Sadia Butt, Muhammad Zubair Yousaf, Muhammad Farooq Sabar

**Affiliations:** 1 National Centre of Excellence in Molecular Biology, 87-West Canal Bank Road Thokar Niaz Baig, Lahore-53700, University of the Punjab, Lahore, Pakistan

## Abstract

**Background:**

Pakistan is a highly endemic area for hepatitis E virus (HEV) infection. The aim of the current study was to isolate and characterize strains of HEV in two mini outbreaks.

**Results:**

RNA was extracted and reverse transcribed to cDNA. Nested PCR was done for the detection of HEV RNA. The positive bands were eluted, cloned in TA vector and sequenced in both directions using genetic Analyzer (Applied Biosystem Inc, USA). Phylogenetic analysis was done using MEGA4 software. We isolated two new HEV genotype-1 strains from Lahore, Pakistan, based on cloning and sequencing of ORF2 region.

**Conclusions:**

Our study suggest that both the human HEV strains were closely related to the Sar-55 but different from the Abb-2B and 87-Pakistan-B HEV isolates sharing 88-91% sequence identity to Pakistani isolate Sar-55. These results indicated that Sar-55 is the main endemic HEV strain in various areas of the country.

## Background

Hepatitis E virus (HEV) is the causative agent of hepatitis E disease that is one of the six known types of human viral hepatitis. It is endemic in many developing countries including Pakistan and is an important public health disease in Asia, Africa and Mexico [1-3]. Hepatitis E is food-borne/water-borne hepatitis as the disease is primarily transmitted by the fecal-oral route via contaminated water or food. The overall mortality rate is generally low (< 1%), however, it has reported to be severe and mortality rates can be as high as 20-25% in pregnant women [4-6]. The genome of HEV is approximately 7.2-kb, and contains a single-stranded, positive sense RNA molecule [7,8]. There are three open reading frames and a short noncoding region each at the 5' and 3' end [9,10]. ORF1 is located towards the 5' end and encodes nonstructural proteins; ORF2, which lies at the 3' end of the genome, encodes the viral capsid protein; ORF3 partially overlaps both ORF1 and ORF2. The ORF3 protein has a cysteine rich region near its amino terminus and has been shown to bind viral RNA and enter into a complex with the capsid protein [10,11].

There are at least four major genotypes of HEV and these four types comprise a single serotype[12]. Extensive diversity among HEV isolates has been reported from patients with acute HEV hepatitis in Pakistan, China and Taiwan [2,12]. It has already been reported that manifold HEV genotypes could co-circulate in the same region and; dissimilar genotypes of HEV could exist in one patient. In Pakistan at least three isolates Sar-55(87-Pakistan-A), Abb-2B(88-Pakistan-2B) and 87-Pakistan-B of HEV have been characterized so far from human patients with acute hepatitis E [2,12,13]. All these three strains belonged to HEV-1 genotype and the nucleotide sequences of these three HEV isolates show about 90% homology. So far genotype 1 HEV has become the dominant cause of hepatitis E disease in Pakistan [11,12].

To our knowledge, no HEV isolate has been characterized from Lahore, Pakistan to date. In this regard we examined two HEV isolates from human patients with acute hepatitis E in two different mini-outbreaks and determined its ORF2 gene sequence. Our study reveals that both the HEV strains were HEV-1 genotype and are divergent from other known strains of HEV-1 strains, confirming that HEV-1 is the predominant genotype of HEV in Pakistan even genetic variation exists in different HEV strains.

## Results

### Demographic characteristics of tested patients

The demographic characteristics of patients are shown in table 1. Majority of the patients were males and their age ranges from 3-20 years.

**Table 1:** Demographic characteristics of treated patients (N = 23)

*S. No*.	*Characteristics*	*No. of Patients*	*Percentage*
1	*Sex*		
	Male	13	57
	Female	10	43
2	*Age range-years*		
	Up to 20	3	14
	Above 20	20	87
3	*Area group*		
	Attock	10	43
	Lahore	12	52
	Gujranwala	1	4
4	*Symptoms*		
	Present	19	82
	Absent	4	17
5	*History of other hepatitis*		
	Anti-HAV IgG	0	0
	HBV HBsAg	0	0
	Anti-HCV	0	0
	Anti-HDV	0	0

### PCR Primer Designing

Table 2 shows the names, sequences, sizes and nucleotide positions of the designed primers. Primer set of number 9 &10 and its nested set primer 11 &12 were found the more sensitive and more specific in all the primer sets. These two sets of HEV primers from the ORF2 gene region were used for the rest of study analysis such as detection and sequencing of Pakistani HEV isolates.

**Table 2:** Names, Sequences, Sizes and Nucleotide positions of primers designed for HEV genotyping assay

*Sr. No*.	*Name*	*Sequence (5'-3')*	*Size (nt.)*	*Nucleotide Position*
1	HEL	GGCCACCTCTGGTCTTGTTA	20	5932-5952
2	HER	GCCGTAAGTGGACTGGTCAT	20	6562-6582
3	HNL	GTCTCCCGTTACTCCAGCAC	20	6080-6100
4	HNR	GGTGAGAGAAAGCCAAAGCA	20	6550-6530
5	RfF1	GCCGAGTATGACCAGTCCA	19	6577-6595
6	RfR1	ACAACTCCCGAGTTTTACCC	20	7127-7107
7	RfF2	AATGTTGCGACCGGCGCGC	19	6649-6668
8	RfR2	TAAGGCGCTGAAGCTCAGC	19	7098-7079
9	OS	AATTATGCCTCAGTACTCGGAGTTG	25	5711-5732
10	OAS	CCCTTAGTCCTTGCTGACGCATTCTC	26	6419-6441
11	IS	GTTAATGCTTCTGCATATCATGGCT	25	5996-6017
12	IAS	AGCCGACGAAATCAATTCTGTC	22	6322-6343

**Figure 1:**
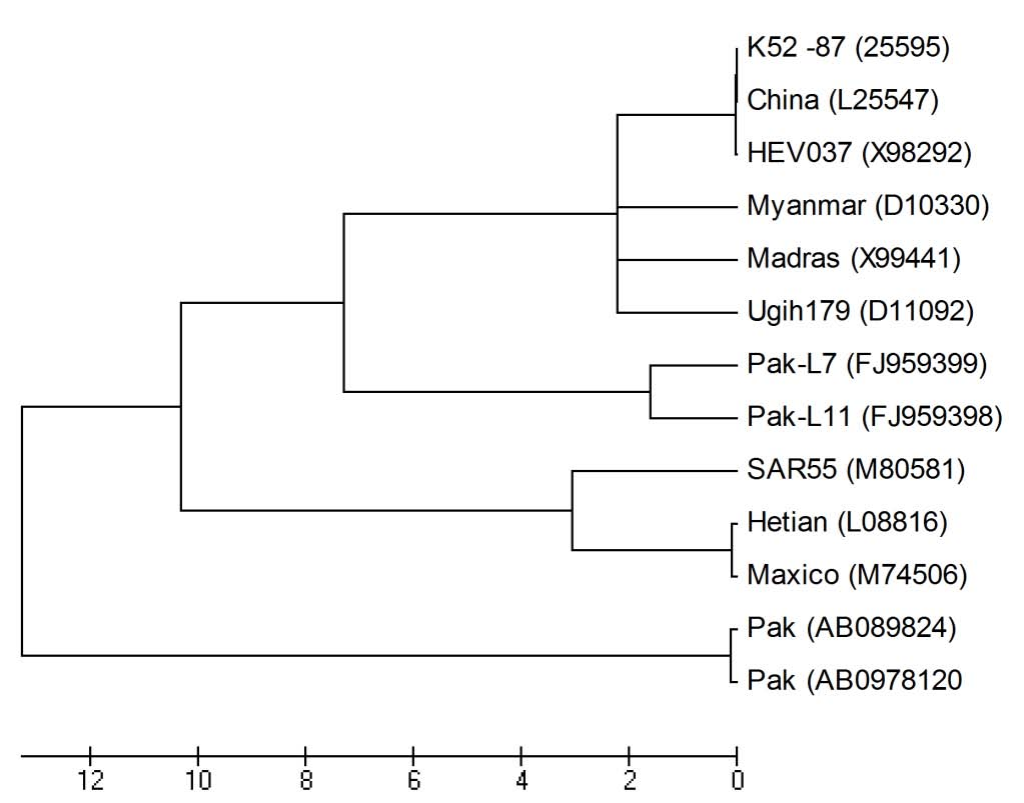
**Phylogenetic analysis based on the ORF2 sequence of both the isolates in current study and other HEV genotypes isolated from different regions of the world, using the neighbor-joining method and evaluated using the interior branch test method with Mega 4 software**. Percent bootstrap support is indicated at each node. The scale bar represents nucleotide substitutions per base. GenBank accession numbers of sequences used for phylogenetic analysis are given in parenthesis.

Figure 1 depict the phylogenetic tree. Three Pakistani HEV-1 genotype strains available in GenBank data base were used as reference sequences in the analysis.

## Discussion

Specific HEV PCR bands were observed in two patient's samples. Our both HEV infected patients were adults with ages of 39 years and 33 years that support the previous studies. In both case the infection was acquired through fecal-oral route. It has already been reported that in Pakistan, HEV remains highly endemic, mainly affecting the adult population. A number of mini-epidemics have been reported previously from Pakistan and all of these appear to have been due to fecal contamination of the water supply [14]. The 348-bp DNA band specific for the HEV ORF2 gene was excised from the gel, purified with the AxyPrep DNA Gel Extraction kit (Axygen, USA) and cloned into TA-Vector (Invitrogen, USA). Both strands of the inserted DNA amplicons were sequenced in a DNA analyzer (Applied Biosystems 3730 DNA Analyzer; Invitrogen, USA). The consensus sequences were made for both the isolates. The sequenced portion of ORF2 gene of both samples was submitted to NCBI GenBank data base. In order to explore the evolutionary association of the HEV isolates sequenced in the current study with other Pakistani HEV genotype 1 isolates and those isolated from different regions of the world, sequences were aligned using MegAlign program in the DNASTAR software package. Using the Mega 4 software http://www.megasoftware.net/ the phylogenetic tree was constructed.

**Figure 2:**
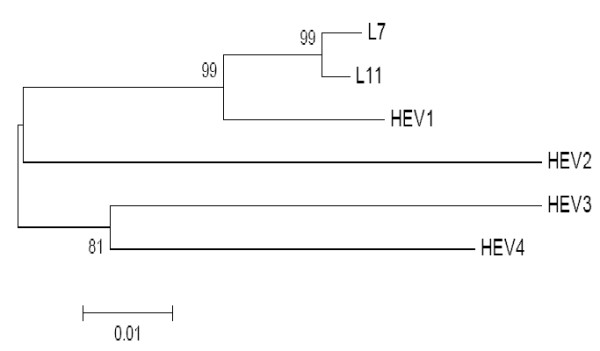
**Phylogenetic analysis based on the complete ORF2 sequence of the isolate in this study and other HEV genotypes 1-4, using the neighbor-joining method and evaluated using the interior branch test method with Mega 4 software**. Percent bootstrap support is indicated at each node. The scale bar represents nucleotide substitutions per base. GenBank accession numbers of sequences used for phylogenetic analysis were: HEV type 1 (M80581), type 2 (M74506), type 3 (AB089824) and type 4 (AB097812).

Pakistani HEV isolates L-7 and L-11 shared 92% sequence identity to each other and is closely related to HEV genotype 1 Pakistani isolate Sar-55 than to other genotypes. The L-7 isolate shared 88% sequence identity and L11 had 90% sequence identity to Pakistani isolate Sar-55. OFR2-based sequences indicated that the two isolates in the present study shared 88-91% identities and 9-12% variation with the other Pakistani HEV-1 genotype, and the maximum sequence identity (91%) with another. Phylogenetic analysis show that both the strains isolated in the current study closely clustered with other HEV Pakistani genotype 1 strains from human, forming a subgroup. Though, the isolated two HEV strains come from Lahore that is geographically far area from other areas from which the HEV-1 strains were isolated initially, they still shared about 88-91% nucleotide homologue with each other, suggesting they may come from a common source where they may emerged from a single isolate. Fecal-oral transmission of HEV genotype 1 may be involved among human in this region. Figure 2 show the phylogentic tree of Pakistani HEV isolates sequenced in the present study with the representative number of sequences for each HEV genotype selected from the GenBank database. The accession numbers of sequences used for phylogentic analysis were: HEV type 1 (M80581), type 2 (M74506), type 3 (AB089824) and type 4 (AB097812).

## Conclusions

Both HEV isolates were related to Sar-55 but unlike from the Abb-2B and 87-Pakistan-B. This leads to conclusion that Sar-55 is the main endemic HEV strain in various areas of the country.

## Methods

### Study design and data Collection

A prospective study was designed and carried out in March 2007 (First mini-outbreak) and December 2007 (second mini-outbreak) to determine the seroprevalence, molecular epidemiology and genotype determination of HEV infection in two mini-outbreaks of hepatitis E virus infection in the city of Lahore, Pakistan. Majority of the subjects were poor, less educated and were more exposed to some of the important established risk factors for various infections particularly more exposed to viral infections. The studied patients were from 20 years to 30 years. The contact was established with doctors or directly with the patients. They were explained the purpose and objectives of the present study. Those who were voluntarily agreed to participate in the study were asked for written informed consent, following which a printed questionnaire was filled from each participant before the collection of blood sample. The questionnaire contained information on name, age, and sex, and socio-economic status, potential risk factors for viral transmission, educational level and eating habits. The questionnaire also included information about any history of jaundice, diagnosis of HAV, HCV or HBV in family.

### Blood Samples Collection

Blood samples (5-10 ml) were collected into tubes from all the enrolled subjects, serum was separated from each sample within 6 hours, aliquot into three 2.0 ml tubes and stored at -70°C. One aliquot was tested for antibody to HEV and HAV by ELISA, routine biochemical liver tests; second aliquot was used for HEV RNA PCR and third was used for sequence analysis.

### Liver function tests and Enzyme-Linked Immunosorbent Assay (ELISA)

All blood samples were tested for routine biochemical liver function tests (LFTs) such as alanine aminotransferase (ALT), aspartate aminotransferase (AST), alkaline phosphatase and bilirubin. Blood samples were also screened for the detection of antibodies against Hepatitis A (IgM), hepatitis B (HBsAg), anti-HCV, HEV (both IgG & IgM) using ELISA kit techniques (DRG Diagnostics, Germany).

### Primer design, detection of ORF2 capsid protein (ORF2) Gene of HEV

For the detection and sequencing of HEV RNA, total 12 primers capable of detecting HEV strains with significant sequence variations were designed based on a multiple sequence alignment of the ORF2 genes of 18 different known strains of human HEV including the three prototype Pakistani strains of HEV using Primer3 program http://bioinformatics.weizmann.ac.il/cgi-bin/primer/primer3.cgi.

Briefly, total RNA was extracted from 100 μl serum samples using Gentra RNA Isolation Kit (Life Technologies, USA). After cDNA synthesis, two rounds of PCR amplifications were done using outer primers 9&10 (First round) and nested primers 11&12 (second round). The PCR products were analyzed in a 1.5% agarose gel.

### Gel Elution

Template preparation for cloning and sequencing of HEV RNA positive samples were carried out with ORF-2 primers using nested RT-PCR. The PCR products were run on 2% agarose gel prepared in 0.5× TBE buffer and purified in a column with a gel extraction kit (QIAGEN, Valencia, California) according to the kit protocol.

### Cloning of ORF2 Gene into PCR 2.1 Vector

The PCR product of ORF2 gene was ligated into the PCR 2.1 vector using TA Cloning Kit (Invitrogen, USA). The kit protocol was followed for ligation and the reaction mixture was incubated at 14 °C for overnight. Heat Shock Transformation method was done by mixing the ligation reaction product (DNA construct) with 100 μl competent TOP 10 cells (Invitrogen). The reaction mixture was kept on ice for 30 minutes following by heat shock at 42°C for 30 seconds. After 3 minutes incubation on ice, 800 μl of LB media was added and again the cells were incubated at 37 °C with shaking for 50 minutes. The white- blue selection method was used for transformants selection. After incubation at 37°C the transformants were spread on agar plates containing Ampicilin and Tetracycline, X-Gal (40 mg/μl) and IPTG (mg/μl). Next day blue and white colonies were observed. The blue colonies contained self ligated PCR 2.1 vector while the white colonies contained desired fragment in PCR 2.1 vector. So, white colonies were selected for further investigation.

### Plasmid DNA Isolation (Plasmid Miniprep) and confirmation of cloning

Plasmid DNA was isolated by alkaline lyses and using JET quick plasmid miniprep spin kit (Genomed, Fermentas) following manufacturer's protocol. To confirm the insert in PCR 2.1 vector, PCR was run with gene specific primers using plasmid DNA as template and then PCR product was sequenced. Restriction digestion of PCR 2.1 vector was also done with EcoR1 (Fermentas) for clone confirmation.

### DNA Sequencing

The purified DNA was used as templates for sequencing PCR in the Big-Dye Terminator cycle sequencing ready reaction kit (Applied Biosystems). Samples were analyzed on an automated sequencer (ABI PRISM 3100 genetic analyzer; Applied Biosystems). Products were sequenced from both strands to get consensus sequences.

### Phylogenic Analysis

Pakistani HEV isolates sequenced in the present study were aligned with the representative number of sequences for each HEV genotype selected from the GenBank database with the help of the Multalign program. Pairwise comparisons for percent nucleotide homology and evolutionary distance were made. The phylogenetic analyses of isolates were performed with MEGA 4.0 software. Jukes-Cantor algorithms were utilized, and phylogenetic trees were constructed by the neighbor-joining method. The reliability of different phylogenetic groupings was evaluated by using the bootstrap-resembling test from the MEGA program (1,000 bootstrap replications).

## Competing interests

The authors declare that they have no competing interests.

## Authors' contributions

MI conceived the study, participated in its design and coordination and gave a critical view of manuscript writing. TI performed the work. AH and LA participated in results analysis. MA, SB, MZY, and MFS participated in sample collection and demographic data. All the authors read and approved the final manuscript.
